# Localized surface plasmon resonance in deep ultraviolet region below 200 nm using a nanohemisphere on mirror structure

**DOI:** 10.1038/s41598-021-84550-w

**Published:** 2021-03-04

**Authors:** Kohei Shimanoe, Soshi Endo, Tetsuya Matsuyama, Kenji Wada, Koichi Okamoto

**Affiliations:** Department of Physics and Electronics, Osaka Prefecture University, 1-1 Gakuen-cho, Naka-ku, Sakai-shi, Osaka, 599-8531 Japan

**Keywords:** Nanoscience and technology, Optics and photonics

## Abstract

Localized surface plasmon resonance (LSPR) was performed in the deep ultraviolet (UVC) region with Al nanohemisphere structures fabricated by means of a simple method using a combination of vapor deposition, sputtering, and thermal annealing without top-down nanofabrication technology such as electron beam lithography. The LSPR in the UV region was obtained and tuned by the initial metal film thickness, annealing temperature, and dielectric spacer layer thickness. Moreover, we achieved a flexible tuning of the LSPR in a much deeper UVC region below 200 nm using a nanohemisphere on a mirror (NHoM) structure. NHoM is a structure in which a metal nanohemisphere is formed on a metal substrate that is interposed with an Al_2_O_3_ thin film layer. In the experimental validation, Al and Ga were used for the metal hemispheres. The LSPR spectrum of the NHoM structures was split into two peaks, and the peak intensities were enhanced and sharpened. The shorter branch of the LSPR peak appeared in the UVC region below 200 nm. Both the peak intensities and linewidth were flexibly tuned by the spacer thickness. This structure can contribute to new developments in the field of deep UV plasmonics.

## Introduction

Plasmonics has recently attracted substantial attention in both science and engineering. In particular, localized surface plasmon resonance (LSPR) has been used in a wide range of applications, such as imaging^1^, sensing^2–6^, light-emitting devices^7–9^, and solar cells^10,11^. SPR has been implemented for efficient light emission from semiconductor light-emitting diodes (LEDs)^12^. Deep ultraviolet LEDs (DUV-LEDs) offer various applications, such as sterilization, processing technology, and optical memory, and exhibit high efficiency. Furthermore, the development of DUV-LEDs has been attracting increasing attention because DUV light sources such as excimer lasers are very large and uneconomic^13–15^. In 2010, we succeeded in improving the efficiency of 260 nm DUV emissions from AlGaN/AlN quantum wells (QWs) by using an Al thin film^16,17^. We found that the internal quantum efficiencies and emission rates were increased by the exciton–SP resonance. A similar method was used to enhance the near-UV and UV emission around 360,300 nm from AlGaN/GaN-based QWs^18^ and LEDs^19,20^. However, in these studies, the peak wavelength of the SP resonance could not be tuned, because Al thin films were used for the SP propagation type. One purpose of this study was to tune the LSPR peak wavelength to extend our proposed method of light emission enhancements to all UV wavelengths. In recent years, LSPR has been applied extensively in the UV region, and it is known that Al is a typical plasmonic metal in the UV region^21^. Al is abundant on earth and offers the advantage of being inexpensive. Ga is another plasmonic metal in the UV region^22^. Ag and Au nanoparticles are easily prepared by chemical methods or thermal annealing to obtain and tune LSPR spectra. However, it is difficult to fabricate nanoparticles with Al using such bottom-up methods. Thus, in many cases, top-down nanofabrication technologies such as electron beam drawing have been used for UV plasmonics using Al^23–26^.

In this study, we obtained and tuned the LSPR in the deep ultraviolet (UVC) region with Al nanohemisphere structures fabricated by means of a simple method using a combination of vapor deposition, sputtering, and thermal annealing without top-down nanofabrication technology such as electron beam lithography. To achieve flexible tuning of the LSPR in a much deeper UVC region below 200 nm, a nanohemisphere on a mirror (NHoM) structure^27^ was designed and evaluated by finite-difference time-domain (FDTD) calculations. Thereafter, Al and Ga structures were fabricated on a substrate by resistance heating evaporation and subsequent thermal annealing to obtain and tune the LSPR spectra in the UVC region. As a result, we successfully extended and tuned the LSPR in the DUV region below 200 nm, which has not been reported previously.

## Methods

The FDTD simulations were conducted using commercial software (Poynting for Optics, Fujitsu, Japan). A periodic boundary condition was set in the X and Y directions, whereas an absorbing boundary condition was set in the Z direction. A pulsed light composed of a differential Gaussian function with a pulse width of 0.5 fs at an x-polarized electric field of 1 V/m was used as excitation. The peak position on the excitation pulse spectrum was approximately 600 THz (500 nm wavelength). The dielectric function of the Al was approximated by the Drude formula based on the values reported by Rakić^28^. The refractive index (*n*) of the Al_2_O_3_ was set to 1.7 without dispersion. A nonuniform mesh with a grid size of 0.5–5 nm was used.

All of the metal nanoparticles and NHoM structures were fabricated on a sapphire substrate (0001). The Al nanoparticles were prepared by forming an Al film with a thickness of 5 or 10 nm by resistance heating evaporation, and subsequent thermal annealing at 420 °C and 475 °C for 10 min in an electric furnace under a nitrogen atmosphere. In a similar manner, the Ga nanoparticles were prepared by forming a Ga film with a thickness of 5, 10 or 15 nm and subsequent heating at 300 °C for 10 min. The NHoM structure was formed by depositing Al (50 nm) by means of resistance heating evaporation and Al_2_O_3_ with a thickness of 3, 5, or 10 nm thickness by atomic layer deposition. The surface morphology was investigated by atomic force microscopy (AFM) (NanoWizard, Bruker – JPK Instruments AG).The transmission and reflection spectra were monitored using a UV–visible spectrometer with a reflectance measurement attachment (5° incident angle, Shimadzu UV-1800, Japan), and were converted into extinction spectra.

## Results and discussion

### Design and theoretical analysis

**Figure 1:**
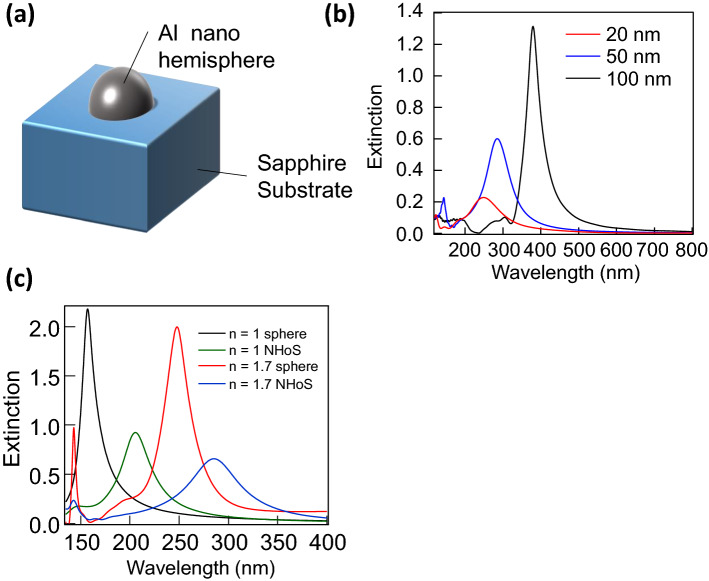
Extinction spectra of spherical and hemispherical Al nanostructures calculated by the FDTD method with various surrounding environment. (**a**) Schematic of Al nanohemisphere fabricated on sapphire substrate. (**b**) Calculated extinction spectra of Al nanohemisphere with diameters of 20, 50, and 100 nm. (**c**) Calculated extinction spectra of Al nanospheres with diameter of 50 nm in medium with refractive index of 1 or 1.7 and Al nanohemispheres with a diameter of 50 nm on substrate with refractive index of 1 or 1.7.

Figure 1 presents the sample structure (a) and extinction spectra obtained by the FDTD simulation (b) of the Al nanohemisphere on the sapphire (NHoS), The main peaks in the wavelength range longer than 200 nm were the dipole oscillation modes, whereas the small peaks below 200 nm were the quadrupole oscillation modes of the LSPR spectra. The peak wavelength of the dipole modes was blue-shifted with a decreasing diameter of the nanohemisphere. However, the extinction also weakened as the size decreased. Further, we calculated the extinction spectra for Al nanospheres and compared them with those obtained for Al NHoS. Figure 1c depicts the extinction spectra of the Al nanospheres in media with n = 1 and 1.7. Under the quasi-electrostatic approximation, the largest LSPR is realized when ε_1_ + 2ε_2_ = 0 (Fröhlish condition), where ε_1_ and ε_2_ are the dielectric constants of the metal nanosphere and the surrounding medium, respectively. As the size of the metal nanosphere increases to reach the wavelength value, the quasi-electrostatic approximation ceases to be valid and causes red-shift of the LSPR peak. Owing to the same reason, the LSPR peaks of Al NHoS shown in Fig. 1b exhibited a red-shift with increasing size of the Al NHoS. Fröhlish condition was satisfied at ~ 120 nm and 220 nm in media with n = 1 and 1.7, respectively. These values indicate the shortest wavelength of the LSPR peak for Al nanospheres in respective medium.

Figure 1c shows that the peak wavelengths of the calculated LSPR spectra of the Al nanosphere were 157 and 247 nm in media with refractive indices of 1 and 1.7, respectively. The peak wavelength became longer than the calculated values of the Fröhlish condition owing to the failure of the quasi-electrostatic approximation. Furthermore, the peak wavelength of the LSPR became longer by approximately 50 and 80 nm in the media with refractive indices of 1 and 1.7, respectively, with the shape change from the Al nanosphere to the Al NHoS structure. From these results, it can be observed that the LSPR peak of the nanohemisphere was longer than that of the nanosphere model and was much more susceptible to the refractive index of the substrate. This is likely because the nanohemisphere exhibits the strongest electrical-field oscillation at the equator; that is, on the surface in contact with the substrate. Therefore, it appears to be difficult to generate a strong dipole oscillation mode peak below 200 nm using an Al nanohemisphere. Although the quadrupole modes in the NHoS appeared around 140 nm, as indicated in Fig. 1c, the peak intensities were very small.

**Figure 2:**
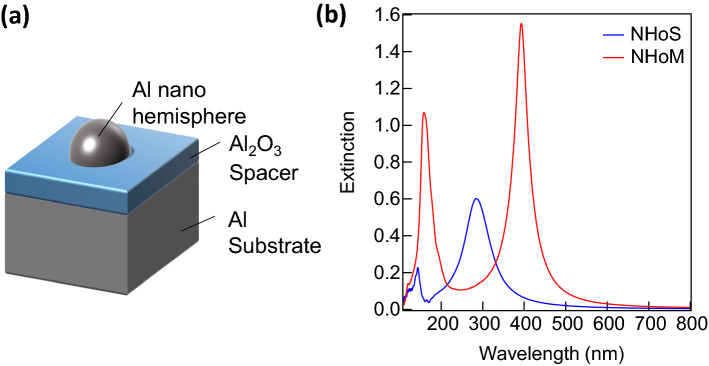
Extinction spectra of the NHoS and NHoM structures calculated by the FDTD method. (**a**) Schematic of NHoM structure. (**b**) Calculated extinction spectra of Al nanohemisphere with diameter of 50 nm, and NHoM structure with diameter of 50 nm and Al_2_O_3_ spacer of 5 nm. The extinction spectrum of the NHoM structure is clearly visible even at wavelengths below 200 nm.

Figure 2a shows the sample structure of the NHoM, which consisted of a dielectric layer and metal particles on a metal substrate. Figure 2b shows the extinction spectra of the Al NHoS and NHoM structures. The diameter of the Al nanohemisphere and thickness of the Al_2_O_3_ pacer layer were set to 50 and 10 nm, respectively. Two distinct peaks were observed in the NHoM structure compared to the NHoS structure. The significantly weak peak below 200 nm in the NHoS structure became notably strong in the NHoM structure. It was also found that the peaks at long wavelengths were stronger than those in the NHoS structure. Moreover, the peaks at longer wavelengths were red-shifted.

**Figure 3:**
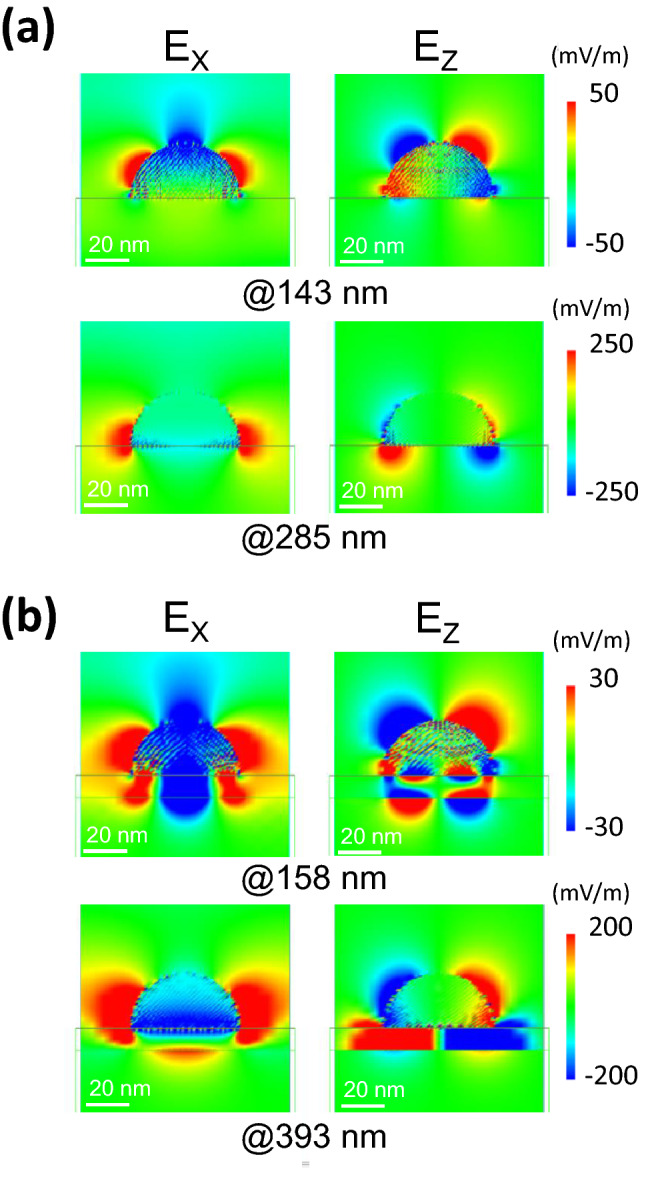
Spatial distribution of electric field for each peak of NHoS and NHoM structures. (**a**) Spatial distribution of electric field around Al nanohemisphere at 143 and 285 nm. (**b**) Spatial distribution of electric field around NHoM at 158 and 393 nm.

Figure 3a presents the spatial distribution of the localized electric field around the NHoS at the peak wavelengths (143 and 285 nm). As mentioned previously, simple dipole and quadrupole modes could be confirmed in the NHoS. Figure 3b shows the spatial distribution of the localized electric field around the NHoM structure at the peak wavelengths (158 and 393 nm). The two peaks of the NHoM structure were the modes owing to the mirror image charges of the metal substrate^27^. The electrical fields of the excitation were included in *E*_x_ as the background because the excitation pulse was x-polarized. These background fields are not particularly visible in this figure because the localized *E*-fields around Al nanoparticles were considerably larger than those of the excitation pulse. The electric field distribution at 393 nm indicates that the charge distribution in the metal nanohemisphere and metal substrate was antisymmetric. The electrons on the surface of the metal film behaved as though they canceled out the external electric field (mirror image charge). In this case, the charge distribution and localized electric field around the metal nanohemisphere appeared similar to the NHoS dipole mode and coupled to the mode on the metal substrate. The electric field distribution at 158 nm demonstrates that the charges at the edge of the hemisphere and metal substrate were antisymmetric, and furthermore, the inner charges of the hemisphere generated propagating SP resonance. The two strong peaks identified in the NHoM structure can be considered to be coupled modes between the dipole and quadrupole modes of the hemisphere and metal substrate. Because the behavior of the charges on one another was antisymmetric, both coupling modes were dark modes, and the width of the peaks was stronger and sharper than that of the NHoS.

**Figure 4:**
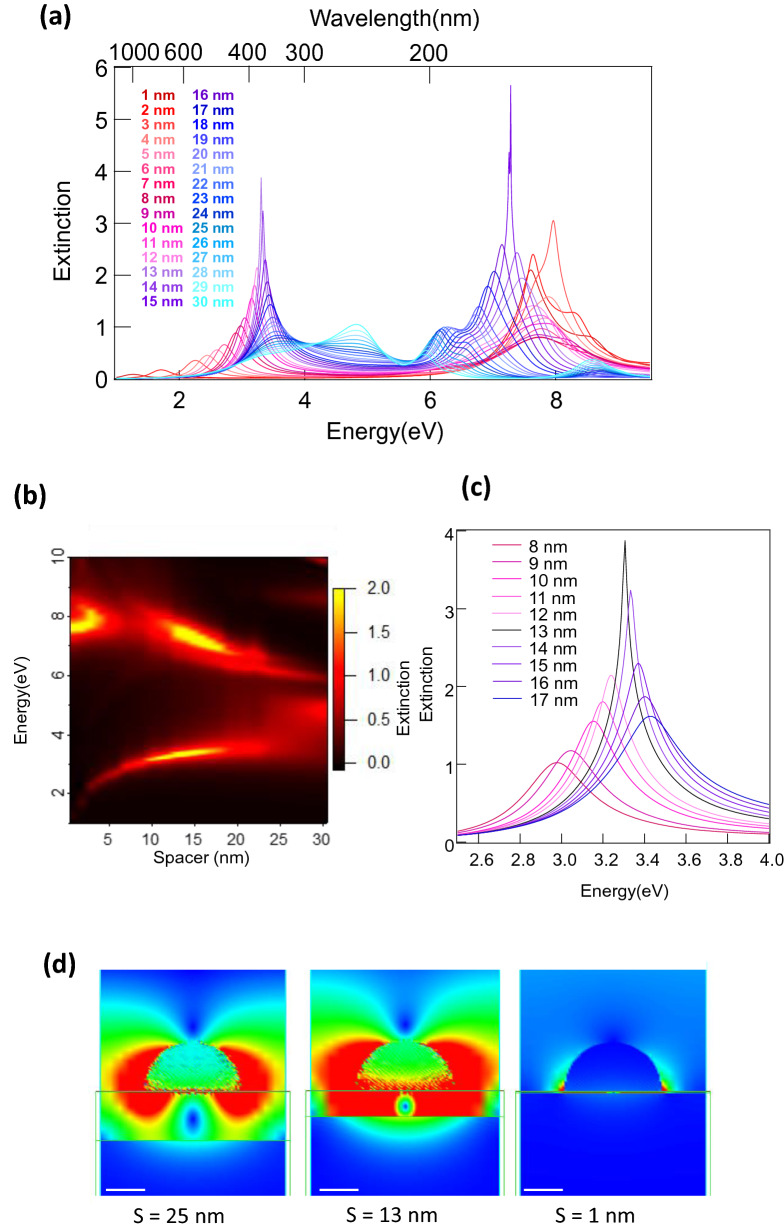
Spacer layer thickness dependence of the extinction spectra of NHoM structure. (**a**) Calculated extinction spectra of Al NHoM structure using Al nanohemisphere with diameter of 50 nm. The Al_2_O_3_ spacer layer thickness ranged from 1 to 30 nm. (**b**) Relationship between peak wavelength of short and long branches and Al_2_O_3_ spacer layer thickness from 1 to 30 nm. (**c**) Long branch of Al_2_O_3_ spacer layer with thickness from 8 to 17 nm. (**d**) Spatial distribution of electric field around NHoM. The spacer layers were 1, 13, and 25 nm.

Figure 4a presents the extinction spectra calculated by the FDTD for the Al NHoM structures with various thicknesses of the Al_2_O_3_ spacer layer ranging from 1 to 30 nm. The diameter of the Al nanohemisphere was 50 nm. Depending on the spacer thickness, several peaks were observed. Figure 4b depicts the relationship between the peak energy level of the short and long branches, and the thickness of the Al_2_O_3_ spacer layer ranging from 1 to 30 nm, in which peak splitting was prominent. This figure indicates that the peak splitting increased as the thickness of the Al_2_O_3_ spacer layer decreased. Two peaks can be clearly observed for a thickness of the Al_2_O_3_ spacer layer in the range from 5 to 20 nm. When the thickness of the Al_2_O_3_ spacer layer was less than 5 nm, the other peak appeared around 8 eV due to the propagation mode. When the thickness increased above 20 nm, the two peaks that appeared for NHoS also appeared around 5 eV and 9 eV for NHoM, and the two prominent peaks that originally appeared for NHoM become weaker. This means that the mode coupling between the metal substrate and metal nanohemisphere weakened with an increasing Al_2_O_3_ spacer layer thickness, and the LSPR should become the same as that for the original nanohemisphere at an infinite thickness. This could be because the skin depth of the localized electric field of the nanohemisphere was approximately equal to its radius. The mode coupling of the NHoM structure became less effective when the distance from the nano-hemisphere to the substrate was longer than the nanohemisphere radius. The results suggest that the NHoM structure could tune LSPR over a wide range from UV to near-infrared. By using the NHoM structure, the tuning of the resonance wavelength could be performed more easily, without changing the size of the Al nanohemisphere, and it was possible that the LSPR could be extended to the UVC wavelength range shorter than 200 nm. We defined the peaks at the long wavelengths (low energy) and short wavelengths (high energy) as the long and short branch modes, respectively. Figure 4c shows the variation in the long branch peaks in the Al_2_O_3_ spacer layer from 8 to 17 nm. The intensity was the strongest and was sharpened when the Al_2_O_3_ spacer layer was 13 nm. This peak was much stronger than that of the Al nanohemisphere on the sapphire substrate. The NHoM structure made it possible to tune the LSPR resonance wavelength easily and to obtain a stronger peak than that of the NHoS.

Figure 4d shows the electric field distribution when the thickness of the spacer layer was 1, 13, and 25 nm. When the spacer layer was 1 nm, the mirror image charge was strongly induced because the nanohemisphere and metal substrate were very close to one another. However, when the spacer was 25 nm, it can be observed that the induced mirror image charge of the metal substrate was weaker than that of the dipole mode of the nanohemisphere. When the spacer layer was 13 nm, both the dipole mode of the nanohemisphere and vibration of the mirror image charge of the metal substrate were stronger than those when the spacer layer was 1 and 25 nm. As mentioned previously, the mirror image charge of the metal substrate and dipole mode of the nanohemisphere exhibited vibrations of the opposite phase, which were apparently similar to the quadrupole mode. It is considered that the darkest mode was obtained by vibrations with the same intensity, and the peak was sharpened at a specific spacer layer thickness. In addition, we found that the propagating mode contributed more when the spacer layer thickness was less than 5 nm, and the interaction between the propagating mode and short branch LSPR mode caused a complicated behavior for a spacer layer thickness in the range of 5–10 nm. This interaction caused the significant change in the peaks around 8 eV shown in Fig. 4b in this region (5–10 nm) of the spacer thicknesses.

Figure S1 in the Supplementary Material shows similar figures to Fig. 4 when using a metallic nanohemisphere with a 100 nm diameter. The behavior was similar to that for the 50 nm-diameter NHoM structure, whereas the peak wavelength was red-shifted when increasing size of the metallic nanohemisphere. The peak splitting of the NHoM with a 100 nm diameter was obvious compared to that with a 50 nm diameter because the skin depth of the electrical field of the LSPR should be deeper and the mode coupling should be much stronger. It is important to note that the spacer thickness at which the LSPR peak was the largest and sharpest for the NHoM with a 100 nm diameter was almost the same as that for NHoMs with 50 nm diameters. The thickness of the spacer layer (metal-to-metal distance) affects the peak intensity more than the size of the nanohemisphere, and a spacer layer thickness of 10 to 15 nm is considered to be appropriate for an NHoM of any size. This may suggest that the strongest and sharpest peaks can be obtained by simply controlling the spacer thickness, even if the size of the fabricated metal nanostructures is somewhat nonuniform and random.

We conclude that the LSPR peak of the NHoM structures can be extended, tuned, and sharpened from the UVC region to the near-infrared region by means of a simple method that only changes the thickness of the Al_2_O_3_ spacer layer. Figure 4a shows that the tunable ranges of the short and long branch are 6–8 eV and 2–4 eV, respectively. These ranges can also be tuned by varying the size of the nanohemispheres; therefore, a wide tunable range from the UVC to the near-infrared region can be obtained by using the NHoM structures with various sizes. In the following section, we describe the experiments in which we fabricated the NHoM structures. It is well known that metal random nanohemispheres are formed by thermal annealing of metal thin films. In many cases, Ag or Au have been used to form nanohemispheres by this method, and the LSPR spectra of the formed structures have been reported in detail^29,30^. We attempted to use a similar method to that of the Al nanohemisphere structures.

### Fabrication and optical measurement

**Figure 5:**
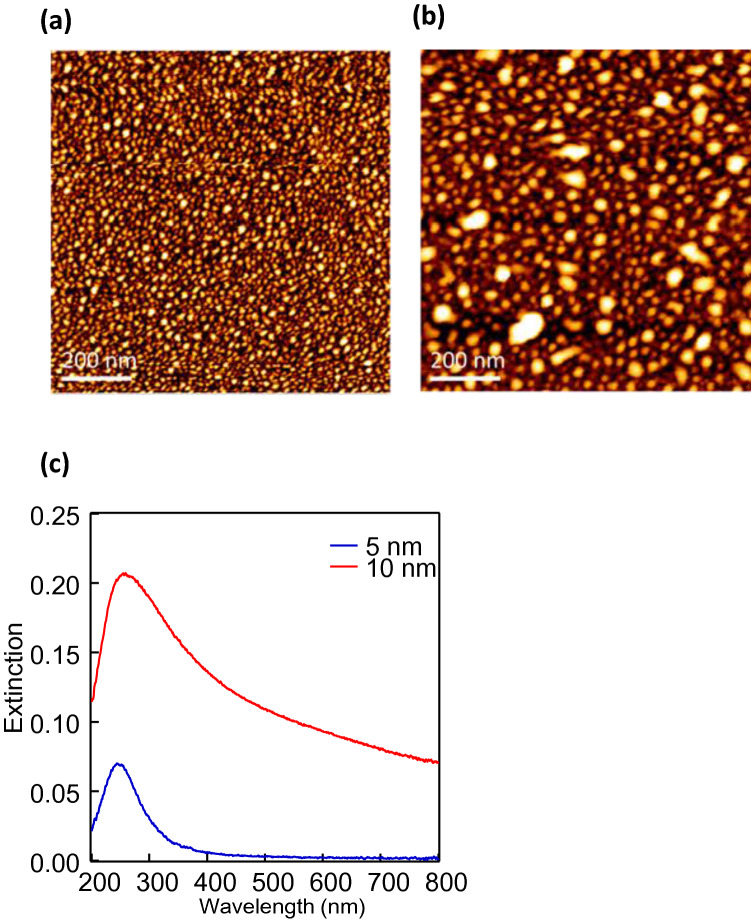
Al nanostructures fabricated by thermal annealing process and its LSPR spectra. AFM images of the Al thin films with (**a**) 5 nm and (**b**) 10 nm after annealing. (**c**) Experimental extinction spectra of 5 and 10 nm Al after annealing.

Figure 5 presents the AFM images of the Al thin films with (a) 5 nm and (b) 10 nm thicknesses following thermal annealing. These images demonstrate that fine particle structures were clearly formed. Particle structures with diameters of approximately 10 to 30 nm were created when the initial film thickness was 5 nm. However, the particle structures became considerably larger when the initial film thickness was 10 nm. Figure 5c presents the observed extinction spectra converted from the transmission spectra of these samples. Clear peaks that could be attributed to the LSPR were observed at 250 and 260 nm when the Al film thickness before annealing was 5 and 10 nm, respectively. These spectra were much broader for the evaporated Al films and became sharper following thermal annealing. The extinction spectrum of the 10 nm Al before heating is also depicted in Fig. S2. This suggests that the Al nanoparticle structures were formed by thermal annealing. The peak was broad, and the peak wavelength was slightly red-shifted for the sample with an initial film thickness of 10 nm. The spectra at the initial film thickness of 10 nm were very broad, particularly in the longer wavelength regions that were not observed in the FDTD calculations. This may suggest that the formed Al nanostructures were not completely separated from one another, and the propagation mode of the SP may also have contributed to the spectra. We attempted to fabricate larger Al nanostructures by increasing the initial film thickness of the Al film above 10 nm; however, no peak derived from the LSPR was obtained. As the wettability of Al against a substrate may be much higher than that of Ag, it is difficult to form nanoparticle structures by thermal annealing. It is also known that Al nanoparticle structures are difficult to form even by chemical synthesis. Although the size could not be adjusted for this reason, we succeeded in fabricating Al particle structures with a diameter of approximately 10 to 30 nm by using a very simple method. The experimental results of the extinction spectra in Fig. 5c were close to the calculated results of the Al nanohemisphere with a 20 nm diameter in Fig. 1b.

**Figure 6:**
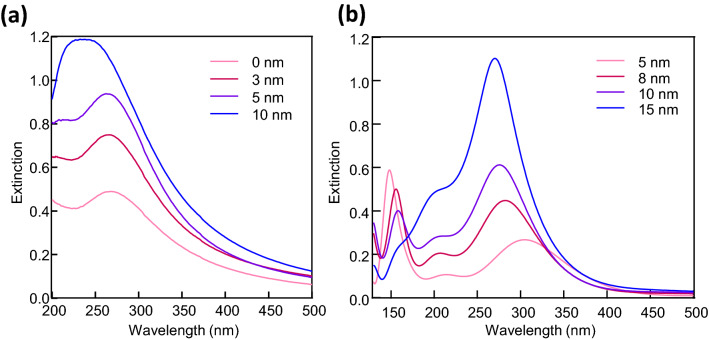
Extinction spectra of the NHoM structure obtained by experimentally and theoretically. (**a**) Experimental extinction spectra of Al NHoM structure. The Al_2_O_3_ spacer layer thicknesses were 0, 3, 5, and 10 nm. (**b**) Calculated extinction spectra of Al NHoM structures with Al nanohemisphere diameter of 20 nm. The Al_2_O_3_ spacer layer thicknesses were 5, 8, 10, and 15 nm.

Figure 6a depicts the experimentally obtained extinction spectra for the NHoM structures with various thicknesses of the Al_2_O_3_ spacer layer. Peak splitting of the LSPR spectra of the Al particle structures was observed for Al_2_O_3_ spacer thicknesses of 0, 3, and 5 nm, for which the peak wavelengths were 267.5, 265.5, and 262 nm, respectively. Although the observed peak shift was slight, a similar peak shift to that of the calculation could be confirmed according to the change in the Al_2_O_3_ spacer layer thickness. We compared these results with the FDTD results of the Al NHoM structure with a diameter of 20 nm. Figure 6b shows the calculated extinction spectra of the Al NHoM structures using the Al nanohemisphere with a 20 nm diameter and Al_2_O_3_ spacer layer thicknesses of 5, 8, 10, and 15 nm. These spectra were similar to the experimental results if we assumed that an oxide film with a thickness of 5 nm was formed at the surface of the Al substrate. As aluminum is known to form a natural oxide layer with a thickness of several nm, we assumed an oxide-layer thickness of 5 nm, which was slightly thicker than normal. Although there is no experimental evidence, we believe that this thickness is possible to realize by a thermal annealing process. Furthermore, these results suggest that the LSPR could be expressed even in the UVC region below 200 nm in the experiment. Peak splitting was not observed in the experiment when the spacer layer was thicker than 10 nm, which was larger than the radius of the fabricated Al particles. The results for the 15 nm spacer layer in the FDTD did not exhibit any peak splitting. The spacer layer was too thick for the 20 nm diameter nanohemisphere to couple between it and the metal film. The experimental results for the 10 nm spacer layer and the FDTD results for the 15 nm spacer layer were both considered to be owing to the thicker spacer layer, which could occur in the original dipole modes. The broad peaks in the experiment were a result of the heterogeneity of the particle structures.

Most importantly, clear peak splittings were observed for 0, 3 and 5 nm spacer layer thicknesses, similar to the calculated results. Unfortunately, our experimental equipment could not detect the wavelength range below 200 nm; however, Fig. 6a shows that the LSPR spectrum exists in deep ultraviolet region, suggesting the presence of a peak in the spectrum in the wavelength range below 200 nm. We achieved the LSPR tuning in deep ultraviolet region below 200 nm using nanohemisphere on mirror structures.

**Figure 7:**
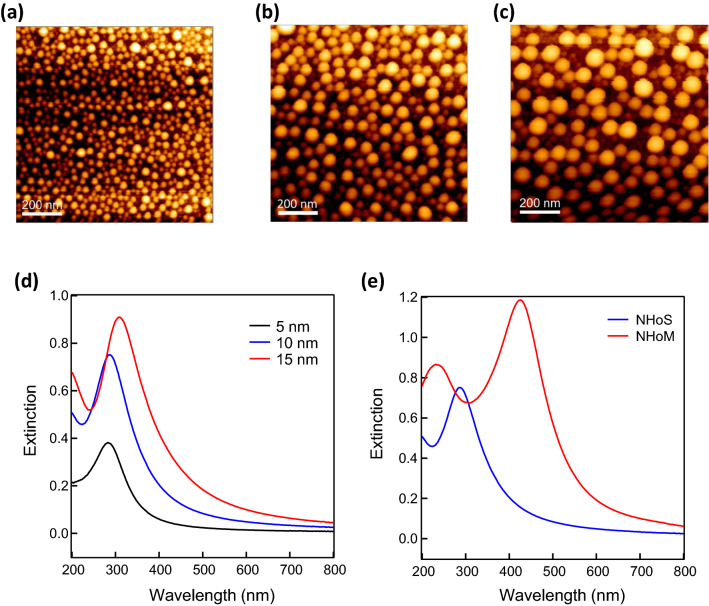
AFM images and extinction spectra of the fabricated Ga nanostructures. AFM images of (**a**) 5 nm, (**b**) 10 nm, and (**c**) 15 nm Ga after annealing. (**d**) Experimental extinction spectra of 5, 10, and 15 nm Ga after annealing. (**e**) Experimental extinction spectra of 10 nm Ga after annealing and Ga NHoM structure. The Al_2_O_3_ spacer layer thickness was 10 nm.

In the experiments using Al, although we observed a tendency for peak splitting, we did not observe two distinct peaks. This is because large Al nanostructures could not be fabricated by our proposed method, and, in addition, our experimental equipment could not detect the wavelength range below 200 nm. To confirm the peak splitting of the LSPR spectra with the NHoM structures in the DUV region more clearly, the same method was used for Ga, which has relatively larger nanostructures and a slightly longer LSPR peak than that of Al. Figure 7 presents the AFM images of the samples with initial film thicknesses of (a) 5 nm, (b) 10 nm, and (c) 15 nm. Relatively larger nanostructures than those of the Al were fabricated, and the size of the nanostructures could be tuned by changing the initial film thickness. This was probably owing to the low wettability of the Ga on the substrate. The grain-like nanostructures were formed before heating and the annealing process resulted in more independent hemispheres. Figure 7d shows the LSPR spectra of the Ga particle structures. The LSPR peak intensities of the Ga particle structures were stronger and the peak wavelengths were longer than those of the Al particle structures. The peak wavelengths of the LSPR spectra after annealing of the 5, 10, and 15 nm Ga were approximately 270, 280, and 310, respectively. This behavior was similar to the results of the FDTD calculations, illustrated in Fig. 1b.

Figure 7e presents the LSPR spectra of the Ga particles and NHoM structures. The NHoM structure was created from the Al metal substrate, Al_2_O_3_ spacer layer (10 nm), and Ga particle structures. The observed peak splitting of the Ga NHoM structure was more obvious than that of the Al NHoM structure. The short and long branches of the split peak were confirmed at approximately 250 and 450 nm, respectively. According to the results of the Ga NHoM structure, the short branch peak should exist below 200 nm for the Al NHoM structure. We succeeded in observing and tuning the LSPR peaks in the UVC wavelength region using the Ga NHoM; however, Ga has not been suitable for electronic and optical device applications because the melting point is too low at 30 °C. Recently, we have successfully obtained stable nanostructures and optical properties by applying protective thin films with thickness of several nm on Ga nanohemispheres, therefore, making Ga useful for device applications^31^. Further, Al is suitable to realize tunable LSPR peaks in the UVC region, even below 200 nm. However, fabricating Al hemispheres larger than ~ 30 nm in diameter by our proposed method remains significantly difficult. Such larger Al NHoM structures can be fabricated using top-down nano-fabrication technologies, which will be reported in the next issue of this paper.

## Conclusions

We have reported that LSPR can be extended to the UVC region even below 200 nm by using an Al NHoM structure. The peak wavelength, bandwidth, and intensity can be tuned flexibly by changing the Al_2_O_3_ spacer layer with these structures by calculations. Peak splitting in the Al NHoM structure was observed experimentally without the use of top-down nanofabrication technologies. We believe that the structures studied can result in the development of a new application field for DUV plasmonics. These structures are expected to be effective in enhancing the emission of DUV light and are applicable to high-efficiency DUV-LEDs. Moreover, this method may be applied to enhance UVC emissions shorter than 200 nm from ultra-widegap semiconductors, such as oxide semiconductors. The proposed structure offers a practical manufacturing method that may be beneficial to engineering applications of LSPR in the UV range.

## Supplementary Information

Supplementary Information 1.

## Data Availability

The data that support the findings of this study are available from the corresponding author upon reasonable request.
