# Supplementary material for: Localized surface plasmon resonance in deep ultraviolet region below 200 nm using a nanohemisphere on mirror structure
Source: Sci Rep. 2021 Mar 4;11:5169. doi: 10.1038/s41598-021-84550-w (PMC7933189; doi:10.1038/s41598-021-84550-w)
Supplement: Supplementary file 1 — Supplementary Information 1. [file 41598_2021_84550_MOESM1_ESM.pdf]

Supplementary Material

Localized surface plasmon resonance in deep ultraviolet region below 200 nm using a nanohemisphere on mirror structure

Kohei Shimanoe, Soshi Endo, Tetsuya Matsuyama, Kenji Wada, and Koichi Okamoto [a)]

Department of Physics and Electronics, Osaka Prefecture University, 1-1 Gakuen-cho, Naka-ku, Sakai-shi, Osaka 599-8531, Japan

Fig. S1 Long branch of extinction spectra of Al NHoM structure calculated using Al nanohemisphere with diameter of 100 nm. The Al_2O_3 spacer layer thickness ranged from 9 to 18 nm.

Fig. S2 Experimental extinction spectra of 5 and 10 nm Al after annealing and 10 nm Al before annealing.

a) okamot@pe.osakafu-u.ac.jp

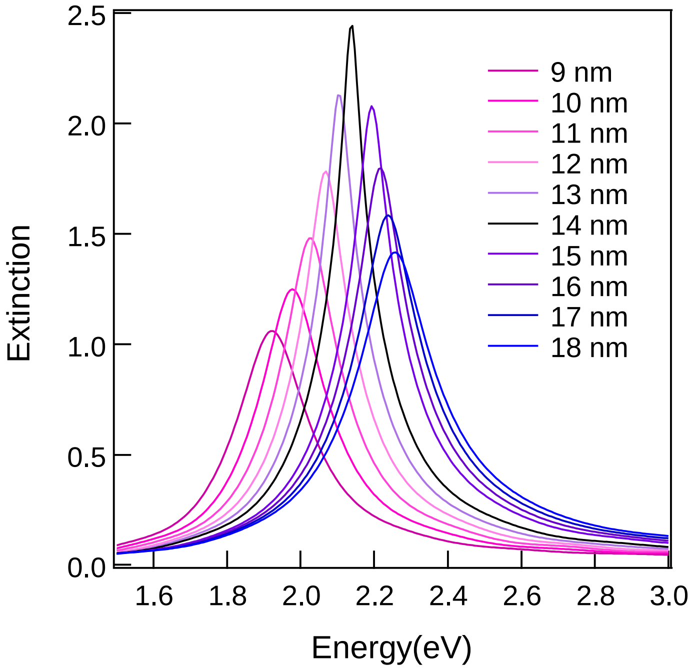

Shimanoe et al. Fig. S1

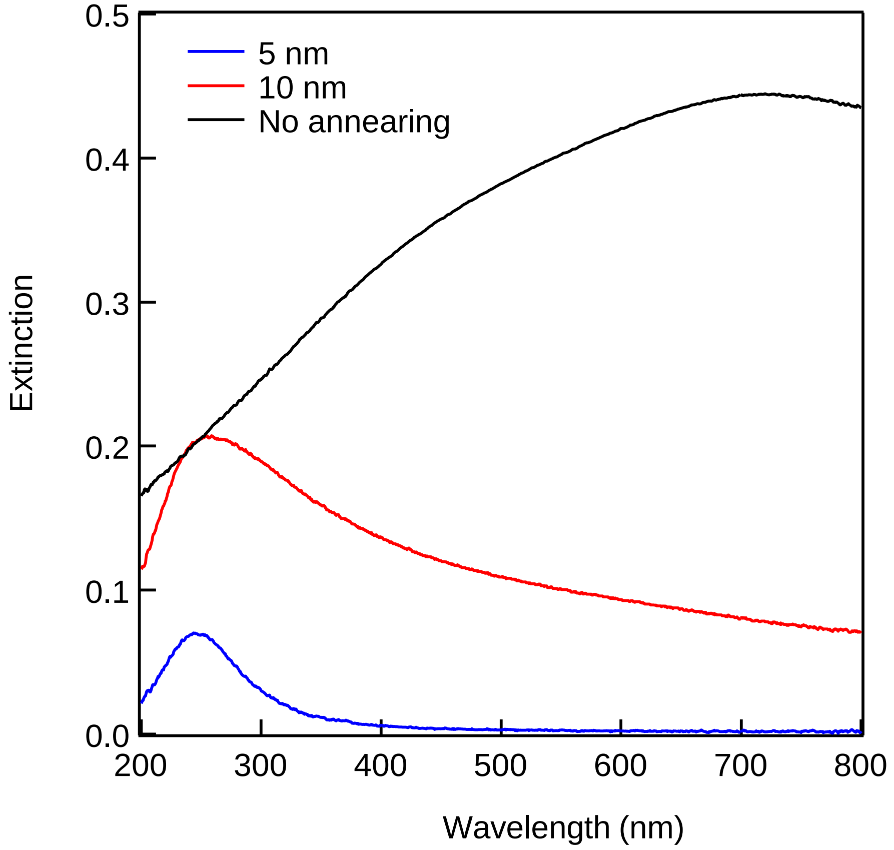

Shimanoe et al. Fig. S2
